# School Mobility and Prospective Pathways to Psychotic-like Symptoms in Early Adolescence: A Prospective Birth Cohort Study

**DOI:** 10.1016/j.jaac.2014.01.016

**Published:** 2014-05

**Authors:** Swaran P. Singh, Catherine Winsper, Dieter Wolke, Alex Bryson

**Affiliations:** a University of Warwick, Coventry, UK; b National Institute of Economic and Social Research, London, UK

**Keywords:** ALSPAC, psychotic symptoms, bullying, social defeat, school mobility

## Abstract

**Objective:**

Social adversity and urban upbringing increase the risk of psychosis. We tested the hypothesis that these risks may be partly attributable to school mobility and examined the potential pathways linking school mobility to psychotic-like symptoms.

**Method:**

A community sample of 6,448 mothers and their children born between 1991 and 1992 were assessed for psychosocial adversities (i.e., ethnicity, urbanicity, family adversity) from birth to 2 years, school and residential mobility up to 9 years, and peer difficulties (i.e., bullying involvement and friendship difficulties) at 10 years. Psychotic-like symptoms were assessed at age 12 years using the Psychosis-like Symptoms Interview (PLIKSi).

**Results:**

In regression analyses, school mobility was significantly associated with definite psychotic-like symptoms (odds ratio [OR] =1.60; 95% CI =1.07–2.38) after controlling for all confounders. Within path analyses, school mobility (probit coefficient [β] = 0.108; *p* = .039), involvement in bullying (β = 0.241; *p* < .001), urbanicity (β = 0.342; *p* = .016), and family adversity (β = 0.034; *p* < .001) were all independently associated with definite psychotic-like symptoms. School mobility was indirectly associated with definite psychotic-like symptoms via involvement in bullying (β = 0.018; *p* = .034).

**Conclusions:**

School mobility is associated with increased risk of psychotic-like symptoms, both directly and indirectly. The findings highlight the potential benefit of strategies to help mobile students to establish themselves within new school environments to reduce peer difficulties and to diminish the risk of psychotic-like symptoms. Awareness of mobile students as a possible high-risk population, and routine inquiry regarding school changes and bullying experiences, may be advisable in mental health care settings.

Nonclinical psychotic symptoms, sometimes referred to as psychosis-like symptoms (PLIKS), are commonly experienced in childhood^1^ and adulthood.^2^ A wealth of research supports the theory that psychosis exists on a continuum^3^ and that subclinical psychotic-like symptoms in childhood significantly increase the risk of psychotic disorder and suicide in adulthood.^1^, ^3^, ^4^ Subclinical and clinical psychosis appear to share similar risk factors,^3^ which suggests that exploring the pathways to PLIKS during childhood may further our understanding of the etiology of psychosis.^2^, ^4^

Well-established psychosocial risk factors for psychosis include migration and urban upbringing.^5^, ^6^ Exposure to adversity such as socioeconomic disadvantage,^7^ family breakdown,^8^ and involvement in bullying^9^, ^10^ have also been associated with the development of this disorder. When considering the associations between urbanicity, residence change, and the development of psychosis in a Danish study, Pedersen and Mortensen^6^ observed that a change in municipality (rather than a change in address within the same municipality) led to an increased risk of psychosis. In Denmark, a change of municipality always leads to a change of school, which led the authors to speculate that the stress of changing schools might explain the increased risk of subsequent schizophrenia, that is, the urban risk may be partly explained by frequent change of schools.

Mobility, especially when linked to school change, may hinder key developmental outcomes. The inevitable breaking of social ties may create psychosocial stress^11^ and increase the risk of antisocial behavior, friendship problems, and bully victimization.^12^ Furthermore, children who are residentially mobile are more likely to experience a range of social adversities, including family dysfunction and financial problems,^13^ and are more likely to belong to ethnic minority groups.^13^ As these factors are also associated with the development of psychosis,^4^, ^7^ we hypothesized that school mobility would be associated with psychosis directly (due to the stress associated with frequent moves), and indirectly as part of a causal chain involving other common risk factors (urbanicity, social adversity, ethnicity, bully victimization).

The aim of the current study was to test whether school mobility is associated with increased risk of psychotic-like symptoms in early adolescence. Specifically, we explored the following questions: Is school mobility independently associated with psychotic-like symptoms when controlling for all other psychosocial risk factors for psychosis? Is school mobility a mediator, that is, a third variable that may help partly to explain the association between prior psychosocial risk (e.g., ethnicity, urbanicity, family adversity) and psychotic-like symptoms? Do peer difficulties (bullying, negative friendships) mediate the association between school mobility and psychotic-like symptoms?

## Method

### Participants

The Avon Longitudinal Study of Parents and Children (ALSPAC) is a UK birth cohort study examining the determinants of development, health, and disease during childhood and beyond. The study has been described in detail elsewhere.^14^ In summary, 14,541 women were enrolled, provided that they were resident in Avon while pregnant and had an expected delivery date between April 1, 1991 and December 31, 1992. A total of 13,978 children (alive at 1 year) formed the original cohort. Ethical approval for the study was obtained from the ALSPAC Law and Ethics committee and the local research ethics committees. Informed consent was obtained from the parents of the children. From the first trimester of pregnancy, parents have completed postal questionnaires about the study child's health and development, while the child has attended annual assessment clinics, including face-to-face interviews and psychological and physical tests.

### Measures

#### Psychotic-like Symptoms

At a mean age of 12.9 years, psychotic-like symptoms were assessed using the semi-structured, face-to-face Psychosis-like Symptoms Interview (PLIKSi). The PLIKSi comprises 12 psychotic symptoms, encompassing hallucinations, delusions, and thought interference over the previous 6 months. The items are derived from the Diagnostic Interview Schedule for Children version IV (DISC-IV) and the Schedules for Clinical Assessment in Neuropsychiatry version 2.0 (SCAN). Trained interviewers rated each item as absent, suspected, or definitely present. The average κ value was 0.72, indicating good interrater reliability.^15^ Two PLIKS variables^9^ were considered: probable/definite ( ≥1 of the 12 PLIKS items was suspected or definitely present), and definite (≥1 of the 12 PLIKS items were definitely present).

### School and Residential Mobility

Mothers reported school mobility when children were approximately 9 years of age. In all, 34 mothers (0.6%) reported no school change; 2,698 (49.5%) reported 1 school change; 2,267 (41.7%) reported 2 school changes; and 446 (8.2%) reported ≥3 school changes. Because of the skewed distribution of responses (very few responses for higher frequencies), we constructed a dichotomous variable: “No school mobility” was coded as 0, 1, or 2 different schools and “school mobility” as 3 or more different schools. As indicated by the distribution of the data, most children experienced 1 or 2 school changes. This reflects the progression through the English school system, typically beginning with nursery or preschool at 4 years of age; reception class at 5 years of age (American equivalent, kindergarten); and primary school from 6 to 11 years of age (American equivalent, elementary school). We used a cut-off point of ≥3 to indicate school mobility as consistent with previously reported definitions of mobile students.^16^

Residential mobility was “mother-reported” when the child was approximately 5, 6, 7, and 8 years of age. Assessment points were selected to match the period defined for school mobility as closely as possible. A total of 3,748 mothers (61.1%) reported no home moves; 1,565 (25.5%) reported 1 home move; 607 (9.9%) reported 2 home moves; and 218 (3.5%) reported ≥3 home moves. Unlike natural school progression changes (e.g., nursery to reception), home moves are not normative as the child progresses through school; therefore, we chose a lower threshold of ≥2 moves to indicate residential mobility.

### Peer Factors

Bully victimization was assessed at 10 years by child report with the Bullying and Friendship Interview Schedule.^17^ Trained psychology graduates asked children about bullying by peers in the past 6 months. Bully victimization was coded as present if the child reported being relationally (e.g., other children not wanting to play with him, spreading rumors about him) and/or overtly bullied (e.g., having been hit or beaten up, having been called nasty names), either frequently (more than 4 times in the last 6 months but less than once per week) or very frequently (at least once per week) at 10 years. Similarly, bully status was coded as present if the child reported relationally (e.g., would not play with others to upset them, told lies/said nasty things about others) and/or overtly bullying others (e.g., hit/beat up others, threatened/ blackmailed others) frequently or very frequently at 10 years. Bully victimization and bully status at 10 years were very highly correlated. To avoid problems with multicollinearity within the path analysis, we collapsed these variables to create involvement in bullying indices: 0 = no involvement; 1 = involvement as a bully or victim; and 2 = involvement as a bully and victim.

Assessment of friendships was based on questions from the Cambridge Hormones and Moods Project Friendship Questionnaire.^18^ Children were asked five questions during clinic sessions, for example, “Do your friends understand you,” or “Do you talk to your friends about problems?” Responses (ranging from 0 to 3) were summed to create a friendship scale from 0 to 15, with 0 denoting the most positive friends score and 15 the least positive.

### Psychosocial Risk Factors

A number of psychosocial risk factors were assessed. Level of urbanicity was ascertained at birth and was coded in line with previous research as 0 = village/hamlet, 1= urban/town.^19^ Multiple social risk factors during pregnancy and from birth to 2 years were assessed using the Family Adversity Index (FAI). The FAI consists of 18 items (e.g., financial difficulties, maternal affective disorder). If an adversity item was reported, it was coded as 1 point, and the points were then summed to derive a total FAI index score for each time point. The 2 FAI scores (pregnancy, 0–2 years) were summed and incorporated into the analysis as a continuous variable. Ethnic background of the child was based on the ethnicity of the mother and her partner. If the mother and/or her partner reported non-white ethnicity, the child was coded as non white.

### Data Analysis

Initial analyses were carried out using SPSS version 19 statistical software. Unadjusted and adjusted associations between psychosocial factors, school mobility, peer difficulties, and subsequent psychotic-like symptoms were computed. Unadjusted associations between psychosocial factors and subsequent school mobility, and school mobility and subsequent peer difficulties, were also computed. Results are reported in odds ratios (ORs) and 95% confidence intervals (CIs) for dichotomous outcomes and β coefficients for continuous outcomes.

Using Mplus version 6, we modeled the pathways via which psychosocial factors and school mobility may be associated with subsequent psychotic-like symptoms. Probit estimation is recommended for path analysis with both categorical (e.g., school mobility) and continuous (e.g., friendship score) endogenous variables.^20^ Probit regression is a log-linear approach analogous to logistic regression, producing similar χ^2^ statistics, *p* values, and conclusions to logit models.^21^ Probit regression coefficients indicate the strength of the relationship between the predictor variable and the probability of group membership. They represent the change in the probability of “caseness” associated with a unit change in the independent variable; thus, it is important to keep the scale of the predictor in mind when interpreting probit coefficients. For example, a probit coefficient of 0.034 indicates that each 1-point increase in the Family Adversity Index resulted in an increase of 0.034 standard deviations in the predicted *z* score of psychotic-like symptoms. Thus, one would expect probit values to be larger for dichotomous predictors, which represent the change from “no caseness” (i.e., no school mobility) to “caseness” (i.e., school mobility) rather than a single value on a continuous scale. The weighted least squares means and variance (WLSMV) estimator (weighted least squares with robust standard errors, mean and variance adjusted) was used, yielding probit coefficients for categorical outcomes and normal linear regression coefficients for continuous outcomes.

## Results

### Descriptive Statistics

Data were available for 6,448 children who completed the Psychosis-like Symptoms Interview (PLIKS)^15^ at the annual assessment clinic at 12 years. Those who were lost to follow-up (54.2%) were more often boys, non white, of low birth weight, born to single mothers of lower educational level, that is, did not obtain O levels (O levels were the standard school-leaving qualification at age 16 in the United Kingdom until recently, when they were replaced by the General Certificate of Secondary Education [GCSEs]), from families living in rented accommodations, and exposed to family adversity (a more detailed analysis is provided by Schreier *et al.*^9^). Those students lost to attrition were also more likely to have moved school ≥3 times, to live in an urban area, and to have been exposed to family adversity (Table S1, available online). A total of 5.6% of adolescents reported definite PLIKS and 13.7% suspected/definite PLIKS. In all, 13.4% had moved home ≥2 times. School and residential mobility were significantly associated with one another. Mobile students were 3.5 (OR = 3.66; 95% CI = 3.08–4.35) times more likely to have moved home ≥2 times (tetrachoric correlation = 0.23).

### Unadjusted and Adjusted Associations Between Psychosocial Factors, School Mobility, Peer Difficulties, and Subsequent Psychotic-like Symptoms

Unadjusted and adjusted associations between psychosocial factors, school and residential mobility, peer difficulties, and subsequent psychotic-like symptoms are reported in Tables 1 and 2. Urban residence, family adversity, residential mobility, school mobility, and peer difficulties were all significantly associated with PLIKS definite and probable/definite symptoms. Combined bully/victim status was strongly associated with PLIKS definite outcome. In multiple logistic regressions, family adversity, school mobility, bullying, and negative friendship score remained significantly associated with definite PLIKS outcome, whereas urbanicity, family adversity, bullying, and friendship score remained significantly associated with PLIKS probable/definite outcome. After adjustment for all other risk factors, school mobility led to an approximately 1.5 times increased risk, and being both a bully and victim of bullying led to an approximately 2.5 times increased risk of definite PLIKS.

**Table 1:** Unadjusted and Adjusted Associations Among Prior Psychosocial Factors, School Mobility, Peer Difficulties, and Definite Psychotic-like Symptom (PLIKS) Status

Risk Factor	PLIKS (Definite) Unadjusted				PLIKS (Definite) Adjusted^a^	
	n	(%)^b^	OR	(95% CI)^c^	OR	(95% CI)^c^
Urbanicity						
Rural (n = 415)	11	(2.7)	[Reference]		[Reference]	
Urban (n = 5,973)	345	(5.8)	**2**.**25**	**(1**.**23–4**.**14)**	1.84	(0.93**–**3.64)
Family adversity^d^						
Mean score	2.69 vs. 3.68^e^		**1**.**09**	**(1**.**06–1**.**13)**	**1**.**09**	**(1**.**05–1**.**13)**
Ethnicity						
White (n = 5,889)	316	(5.4)	[Reference]		[Reference]	
Nonwhite (n = 242)	19	(7.9)	1.50	(0.93**–**2.43)	1.05	(0.56**–**1.97)
Residential mobility						
<2 Moves (n = 5,322)	277	(5.2)	[Reference]		[Reference]	
≥2 Moves (n = 825)	66	(8.0)	**1**.**58**	**(1**.**20–2**.**09)**	1.38	(0.98**–**1.94)
School mobility						
<3 Moves (n = 4,997)	243	(4.9)	[Reference]		[Reference]	
≥3 Moves (n = 446)	37	(8.3)	**1**.**77**	**(1**.**23–2**.**54)**	**1**.**60**	**(1**.**07–2**.**38)**
Peer difficulties						
Bully involvement at 10 years						
None (n = 4,383)	173	(3.9)	[Reference]		[Reference]	
Bully or victim (n = 1,149)	99	(8.6)	**2**.**29**	**(1**.**78–2**.**96)**	**2**.**18**	**(1**.**62–2**.**92)**
Bully and victim (n = 320)	38	(11.9)	**3.28**	**(2**.**62–4.75)**	**2**.**48**	**(1.58–3**.**90)**
Negative friendship at 10 years						
Mean score	2.95 vs. 3.35^e^		**1**.**08**	**(1**.**03–1**.**14)**	**1**.**07**	**(1**.**01–1**.**13)**

Note: OR = odds ratio.

a Multiple regression including all predictors in model.

b Proportion of participants for risk factor (no vs. yes) with 1+ psychotic-like symptoms.

c Boldface type indicates that the 95% CI does not include 1.00.

d Family adversity assessed during pregnancy and birth to 2 years.

e Mean scores reported as independent variables in logistic regression on a continuous scale.

**Table 2:** Unadjusted and Adjusted Associations Among Prior Psychosocial Factors, School Mobility, Peer Difficulties, and Probable/Definite Psychotic-like Symptom (PLIKS) Status

Risk Factor	PLIKS (Probable/Definite) Unadjusted				PLIKS (Probable/Definite) Adjusted a	
	n	(%)^b^	OR	(95% CI)^c^	OR	(95% CI)^c^
Urbanicity						
Rural (n = 415)	28	(6.7)	[Reference]		[Reference]	
Urban (n = 5,973)	846	(14.2)	**2**.**28**	**(1**.**54–3**.**37)**	**2**.**31**	**(1**.**45–3**.**67)**
Family adversity^d^						
Mean score	2.64 vs. 3.38^e^		**1**.**08**	**(1**.**05–1**.**10)**	**1**.**07**	**(1**.**04–1**.**10)**
Ethnicity						
White (n = 5,889)	796	(13.5)	[Reference]		[Reference]	
Nonwhite (n = 242)	37	(15.3)	1.16	(0.81–1.65)	0.93	(0.60**–**1.45)
Residential mobility						
<2 Moves (n = 5,322)	691	(13.0)	[Reference]		[Reference]	
≥2 Moves (n = 825)	142	(17.2)	**1**.**39**	**(1**.**14–1**.**70)**	1.23	(0.97**–**1.55)
School mobility						
<3 Moves (n = 4,997)	642	(12.8)	[Reference]		[Reference]	
≥3 Moves (n = 446)	75	(16.8)	**1**.**37**	**(1**.**06–1**.**78)**	1.24	(0.93**–**1.66)
Peer difficulties						
Bully involvement at 10 years						
None (n = 4,383)	481	(11.0)	[Reference]		[Reference]	
Bully or victim (n = 1,149)	216	(18.8)	**1**.**88**	**(1**.**58–2**.**24)**	**1.79**	**(1.47–2.19)**
Bully and victim (n = 320)	71	(22.2)	**2**.**31**	**(1**.**75–3**.**06)**	**1.90**	**(1.36–2.64)**
Negative friendship at 10 years						
Mean score	2.93 vs. 3.25^e^		**1**.**07**	**(1**.**03–1**.**11)**	**1**.**06**	**(1**.**02–1**.**10)**

Note: OR = odds ratio.

a Multiple regression including all predictors in model.

b Proportion of participants for risk factor (no vs. yes) with 1+ psychotic-like symptoms.

c Boldface type indicates that the 95% CI does not include 1.00.

d Family adversity assessed during pregnancy and birth to 2 years.

e Mean scores reported as independent variables in logistic regression on a continuous scale.

### Unadjusted Associations Between Psychosocial Factors and Subsequent School Mobility

Associations between psychosocial factors and school mobility were assessed. Family adversity (OR = 1.05; 95% CI =1.02–1.09) and ethnicity (OR = 1.78; 95% CI = 1.16– 2.75) were significantly associated with school mobility.

### Unadjusted Associations Between School Mobility and Subsequent Peer Difficulties

School mobility was significantly associated with bully status (OR = 1.47; 95% CI = 1.02–2.14), bully victimization (OR = 1.26; 95% CI = 1.01–1.59) and negative friendship score (β = 0.50; 95% CI = 0.29–0.72).

### Direct and Indirect Associations Between Psychosocial Factors, School Mobility, Peer Difficulties, and Subsequent Psychotic-like Symptoms

**Figure 1:**
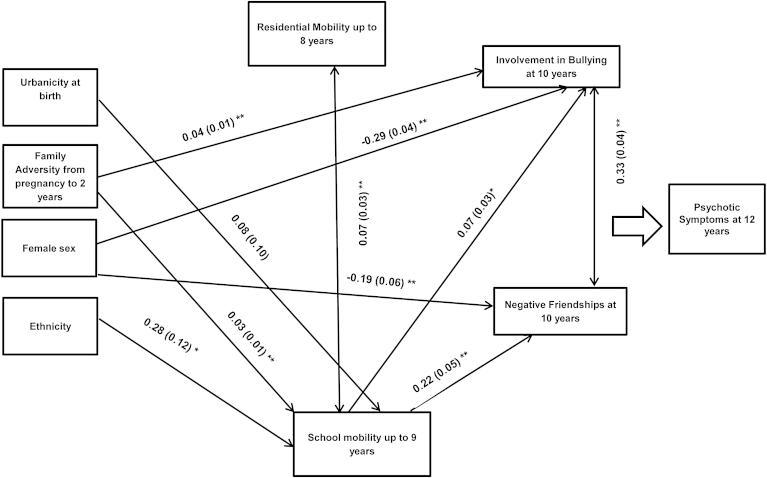
Path model depicting the direct pathways to school mobility and peer difficulties within the final model. Note: Pathways to psychotic outcome not shown for clarity (reported in Tables 3 and 4). ∗Significant at 0.05; ∗∗significant at 0.01.

We conducted 2 path models using definite and probable/definite psychotic-like symptom outcomes. Based on existing literature, in the first path model we incorporated direct associations between all psychosocial risk factors (i.e., family adversity, urbanicity, and ethnicity), sex, school mobility, residential mobility, bullying involvement and subsequent psychotic-like symptoms, and indirect associations from psychosocial risk factors, sex, and school mobility to psychotic-like symptoms (Figure 1 shows direct pathways within the final models). Thus, urbanicity, ethnicity, sex, family adversity, and residential mobility were incorporated as exogenous (independent) variables; school mobility and peer difficulties as independent, mediating and dependent variables; and psychotic-like symptoms as the main endogenous (outcome) variable. The fit indices indicated that there was room for improvement in model fit (χ^2^ = 66.20; *p* < .001; root mean square error of approximation [RMSEA] = 0.026; comparative fit index [CFI] = 0.85). Inspection of the modification indices suggested that incorporating a pathway from family adversity to bullying involvement would improve model fit. As this pathway was consistent with the research literature,^22^ it was incorporated into the final model leading to a considerably improved model fit: definite PLIKS outcome (χ^2^ = 16.57; *p* = .17; RMSEA = 0.008; CFI = 0.99) and probable/definite PLIKS outcome (χ^2^ = 16.43; *p* = .17; RMSEA = 0.008; CFI = 0.99). Bullying involvement was incorporated as an ordinal variable (0 = no involvement; 1= involvement as a bully or victim; 2 = involvement as a bully and victim) consistent with the observed dose–response relationship in the unadjusted analysis. In Mplus, an ordinal variable is treated as a continuous latent variable that exceeds thresholds to give the various outcome categories. One coefficient per ordinal variable is produced. This can be interpreted in the same way as a continuous variable. Direct associations among psychosocial factors, school mobility, and peer difficulties are shown in Figure 1 (pathways to psychotic-like symptoms are not shown for clarity). Family adversity and ethnicity predicted school mobility, whereas school mobility predicted bullying involvement and negative friendship score. Boys were more likely to be involved in bullying and to report negative friendships. Direct and indirect pathways to psychotic-like symptom outcome are shown in Table 3 (definite symptoms) and Table 4 (probable/definite symptoms). Family adversity, urbanicity, and bullying involvement were independently associated with PLIKS definite and probable/definite symptoms. School mobility was independently associated with PLIKS definite symptoms. There was a significant indirect association between school mobility and PLIKS (definite and probable/definite) via bullying involvement, and a significant indirect association between family adversity and PLIKS (definite and probable/definite) via bullying involvement. The indirect associations were of a relatively small magnitude, indicating partial mediation. For example, the indirect effect of school mobility on definite psychotic-like symptoms via bullying involvement was 0.018, whereas the direct association between school mobility and psychotic-like symptoms was 0.108. Therefore, the ratio of indirect effect to direct effect was 0.17, that is, the indirect effect was approximately one-sixth of the size of the direct effect.

**Table 3:** Nonstandardized Probit Coefficients (β) for the Main Direct and Indirect Pathways Among Psychosocial Adversities, School Mobility, Peer Difficulties, and Subsequent Psychotic-like Symptoms (PLIKS) Outcome (Definite)

	Direct to PLIKS Definite			Indirect to PLIKS Definite					
				Via Bullying Involvement			Via School Mobility		
	β	SE	*p*	β	SE	*p*	β	SE	*p*
Family adversity	**0**.**034**^a^	**0**.**009**	**<**.**001**	**0**.**010**	**0**.**002**	**<**.**001**	0.003	0.002	.069
Ethnicity	0.071	0.131	.587				0.030	0.019	.120
Urbanicity	**0**.**342**	**0**.**142**	.**016**				0.008	0.012	.517
Female sex	**0**.**203**	**0**.**057**	**<**.**001**	**−0**.**069**^b^	**0**.**014**	**<**.**001**			
School mobility	**0**.**108**	**0**.**052**	.**039**	**0**.**018**	**0**.**008**	.**034**			
Residential mobility	0.139	0.077	.072						
Negative friendships	0.011	0.013	.380						
Bullying involvement	**0.241**	**0**.**037**	**<**.**001**						

Note: Boldface indicates significant associations. β = probit coefficient; SE=standard error.

a Results reported in probit coefficients: a probit coefficient of 0.034 indicates that each 1-point increase in the Family Adversity Index resulted in an increase of 0.034 SD in the predicted *z* score of psychotic-like symptoms.

b Negative number indicates male sex as variable coded as 1 = male, 2 = female.

**Table 4:** Nonstandardized Probit Coefficients (β) for the Main Direct and Indirect Pathways Among Prior Psychosocial Adversities, School Mobility, Peer Difficulties, and Subsequent Psychotic-like Symptoms (PLIKS) Outcome (Suspected/Definite)

	Direct to PLIKS Probable/Definite			Indirect to PLIKS Probable/Definite					
				Via Bullying Involvement			Via School Mobility		
	β	SE	*p*	β	SE	*p*	β	SE	*p*
FAI	**0**.**030**^a^	**0**.**007**	**<**.**001**	**0**.**008**	**0**.**002**	**<**.**001**	0.002	0.001	.224
Ethnicity	−0.047	0.108	.663				0.014	0.013	.262
Urbanicity	**0**.**451**	**0**.**104**	**<**.**001**				0.004	0.006	.527
Female sex	**0**.**136**	**0**.**043**	.**002**	**−0**.**057**^b^	**0**.**011**	**<**.**001**			
School mobility	0.052	0.041	.201	**0**.**015**	**0**.**007**	.**037**			
Residential mobility	0.121	0.063	.055						
Negative friendships	0.018	0.010	.073						
Bullying involvement	**0**.**202**	**0**.**029**	**<**.**001**						

Note: Boldface indicates significant associations. β = probit coefficient; FAI = Family Adversity Index; SE = standard error.

a Results reported in probit coefficients: a probit coefficient of 0.030 indicates that each 1-point increase in the FAI resulted in an increase of 0.030 standard deviation in the predicted z score of psychotic-like symptoms.

b Negative number indicates male sex as variable coded as 1 = male, 2 = female.

## Discussion

Using data from the ALSPAC cohort study, we explored whether, and how, school mobility might be associated with increased risk of psychotic-like symptoms in early adolescence. First, we found that school mobility is independently associated with an increased risk of psychotic-like symptoms, even when controlling for all other psychosocial risk factors. School change is stressful for students.^23^, ^24^ Psychologically, it can lead to the formation or exacerbation of negative schemata, such as low self-esteem^25^ and external locus of control.^24^ As negative schemata have also been associated with the development of psychotic symptoms,^26^, ^27^, ^28^ such schema may represent 1 mechanism by which school mobility could increase the risk of psychotic-like symptoms. In addition, repeated school change may induce feelings of social defeat (i.e., the negative experience of being excluded from the majority group),^29^ which, if chronic, may lead to sensitization of the mesolimbic dopamine system, and hence heighten the risk of psychotic-like symptoms in vulnerable individuals.^30^

Second, school mobility was also associated with an increased risk of psychotic-like symptoms via bullying involvement, indicating a second “indirect” pathway through which school mobility may be associated with increased risk. Consistent with previous research,^9^, ^31^ we found a significant association between bullying involvement and psychotic-like symptoms; involvement in bullying was the strongest predictor of psychotic-like symptoms, leading to an approximately 2.5 times increased risk. Results here expand on current evidence by highlighting mobile students as an especially “at risk” group for bullying involvement. Consistent with previous research,^32^, ^33^ we found that mobile students were more likely to encounter negative friendships and bullying. Indeed, research suggests that mobile students tend to view themselves as insecure and to have fewer friends than their less-mobile peers.^24^, ^34^ These observations are also consistent with the social defeat hypothesis of psychosis, which has been postulated as the mechanism linking social risk factors to psychosis. Therefore, peer problems may add to psychosocial adversities in a cumulative way, presenting a further source of marginalization, exclusion, and social defeat.^30^

Third, we found that urbanicity, ethnic status, and family adversity were independently associated with psychotic-like symptoms. Consistent with previous research, we found that mobile students were more likely to have experienced family adversity and to be of ethnic minority status,^13^ suggesting that those who experience adversity and marginalization from a young age are more likely to change school more often. However, school mobility was not found to be a mediator of the association between such psychosocial risks and psychotic-like symptoms. Instead, the effects of family adversity were partly mediated by involvement in bullying at school. This confirms previous research that family stresses increase the risk of involvement in bullying^22^ and adverse mental health outcomes, including psychotic-like symptoms.^10^

This study has a number of strengths. We used a large, longitudinal data set, and were able to take into account a number of psychosocial factors associated with school mobility and psychotic-like symptoms. Using path analyses, several pathways to psychotic-like symptoms were quantified while taking into account the time ordering of exposures, enabling us to assess the potential temporal associations between school mobility, other risk factors, and subsequent psychotic-like symptoms.

There are also limitations to this study. Although we controlled for residential mobility, we were unable to distinguish between school moves with and without concomitant home moves. Although many educators believe that school mobility is an inevitable consequence of moving homes, research suggests that approximately 40% of school moves are not associated with residential changes.^23^ Furthermore, we did not control for any pre-existing peer difficulties or individual traits present before the child's first entry into school, which may have contributed to subsequent school mobility^35^ and bullying experiences.^36^ Second, there were missing data, resulting in a reduced sample size. This reduces statistical power and therefore works against our hypotheses, rather than inflating effects.^37^ We found that those who were lost to attrition were more likely to have moved school 3 or more times, to live in an urban area, to be of ethnic minority, and to have been exposed to family adversity. Previous simulations with this longitudinal data resource indicate that selective dropout may underestimate the prevalence of psychiatric disorders but has only a small impact on associations between predictors and outcomes, even when dropout is correlated with predictor variables.^38^ Nevertheless, selective dropout will have reduced the representativeness of our sample. Third, the psychosis outcome referred to symptoms occurring over the previous 6 months only, and for some adolescents, these phenomena may have been transient and self-limiting. However, recent long-term follow-up indicates that psychotic experiences in childhood highly increase the risk of psychosis in adulthood.^4^

Our study demonstrates that school mobility is independently and also indirectly associated with psychotic-like symptoms via bullying involvement. As bullying^35^ and school exclusion^39^ may significantly contribute to student mobility and are also associated with risk factors for psychosis, including social deprivation,^22^, ^40^ ethnicity,^41^ and alienation from mainstream society,^40^ the impact of school exclusion on mental health outcomes may be a fruitful route of inquiry. Although school moves may be unavoidable, involvement in bullying and isolation from peers are amenable to psychosocial interventions^42^ and may be a focus of attention for mobile students. Reports suggest that teachers may lack the time and resources to ensure that mobile students are adequately established within new school environments.^43^ Pilot schemes indicate that the addition of dedicated “mobility support workers” may help mobile students to successfully establish themselves within new school environments,^44^ reducing the risk of bullying involvement and other social difficulties. An awareness of mobile students as a possible high-risk population and routine inquiry regarding school changes and bullying experiences may be advisable in mental health care settings.^45^

**Clinical Guidance**

• School mobility (i.e., moving schools ≥3 times) during childhood may increase the risk of psychotic-like symptoms in early adolescence, both directly and indirectly via increased risk of bullying involvement.
• When assessing young persons with psychotic disorders, clinicians should explore history of school mobility and its psychological/emotional impact, particularly of bullying and marginalization.
• Strategies to help mobile students to establish themselves within new school environments (e.g., through use of mobility support workers) may help to reduce peer difficulties and to diminish the risk of psychotic-like symptoms.

## Figures and Tables

**Table S1:** Dropout Analysis Comparing Those With and Without the Psychotic-like Symptoms (PLIKS) Interview

Characteristic	PLIKS Interview Not Available, n (%)	PLIKS Interview Available, n (%)	PLIKS Interview Not Available vs. Available, OR (95% CI)^a^
School mobility			
No	1,989 (88.5)	4,990 (91.8)	[Reference]
Yes (≥3 moves)	258 (11.5)	446 (8.2)	**0.69 (0.59–0.81)**
Residential mobility			
No	3,846 (87.6)	5,313 (86.6)	[Reference]
Yes (≥2 moves)	545 (12.4)	825(13.4)	1.10 (0.98–1.23)
Urbanicity			
Rural	349 (4.7)	415 (6.5)	[Reference]
Urban	7,065 (95.3)	5,964 (93.5)	**0.71 (0.61–0.82)**
Ethnicity			
White	5,592 (93.8)	5,882 (96.1)	[Reference]
Nonwhite	368 (6.2)	241 (3.9)	**0.62 (0.53–0.74)**
Bullying involvement			
No	853 (71.8)	4,380 (74.9)	[Reference]
Bullied or bully	259 (21.8)	1,147 (19.6)	0.86 (0.74–1.00)
Bullied and bully	76 (6.4)	320 (5.5)	0.82 (0.63–1.06)
Friendship score			
Mean (SD)	2.90 (2.22)	2.96 (2.13)	*p* = .38
Family adversity score	3.43 (3.35)	2.74 (2.99)	***p* ≤ .001**

Note: OR = odds ratio.

a Boldface type indicates that the 95% CI does not include 1.00.
